# Supplementary material for: CFTR Function Restoration upon Elexacaftor/Tezacaftor/Ivacaftor Treatment in Patient-Derived Intestinal Organoids with Rare CFTR Genotypes
Source: Int J Mol Sci. 2023 Sep 26;24(19):14539. doi: 10.3390/ijms241914539 (PMC10572896; doi:10.3390/ijms241914539)
Supplement: Supplementary file 1 [file ijms-24-14539-s001.zip › Legends_supps.pdf]

Supplemental figure S1

Overview of western blots of PDIOs with rare *CFTR* genotypes in the presence of ETI or DMSO (vehicle) treatment with Hsp90 loading control.

Supplemental figure S2

SLA upon 24 hour ELX/TEZ incubation versus relative levels of CFTR C-band (calculated from western blots) for PDIOs with rare *CFTR* genotypes.

Supplemental figure S3

FIS in response to ELX/TEZ/IVA treatment for all individual PDIOs with rare *CFTR* genotypes. **(A)** FIS response in PDIOs with an SLA <41% at the start of the FIS assay, sorted from highest to lowest responder at 0.128 µM forskolin. **(B)** FIS response of PDIOs with an SLA of >41% at the start of the FIS assay.

Supplemental figure S4

FIS at 0.128 µM forskolin versus SLA after 24 hour ELX/TEZ incubation for PDIOs with rare *CFTR* genotypes.

Supplemental table 1

Overview of nonsense variants of F508del/class I PDIOs used.
