# Supplementary material for: CFTR Function Restoration upon Elexacaftor/Tezacaftor/Ivacaftor Treatment in Patient-Derived Intestinal Organoids with Rare CFTR Genotypes
Source: Int J Mol Sci. 2023 Sep 26;24(19):14539. doi: 10.3390/ijms241914539 (PMC10572896; doi:10.3390/ijms241914539)
Supplement: Supplementary file 1 [file ijms-24-14539-s001.zip › table S1_F508del genotypes_revised.pdf]

**Donor ID**	**Allele 1**	**Allele 2**
PDIO 01	F508del	R1162X
PDIO 02	F508del	G542X
PDIO 03	F508del	Y1092X
PDIO 04	F508del	R1162X
PDIO 05	F508del	R1162X
PDIO 06	F508del	E730X
PDIO 07	F508del	Y1092X
PDIO 08	F508del	Y1092X
PDIO 09	F508del	S489X
PDIO 10	F508del	R553X
PDIO 11	F508del	W1282X
PDIO 12	F508del	W1282X
PDIO 13	F508del	E60X
PDIO 14	F508del	W1282X
PDIO 15	F508del	R1158X
