# Supplementary figures and images for: CFTR Function Restoration upon Elexacaftor/Tezacaftor/Ivacaftor Treatment in Patient-Derived Intestinal Organoids with Rare CFTR Genotypes
Source: Int J Mol Sci. 2023 Sep 26;24(19):14539. doi: 10.3390/ijms241914539 (PMC10572896; doi:10.3390/ijms241914539)

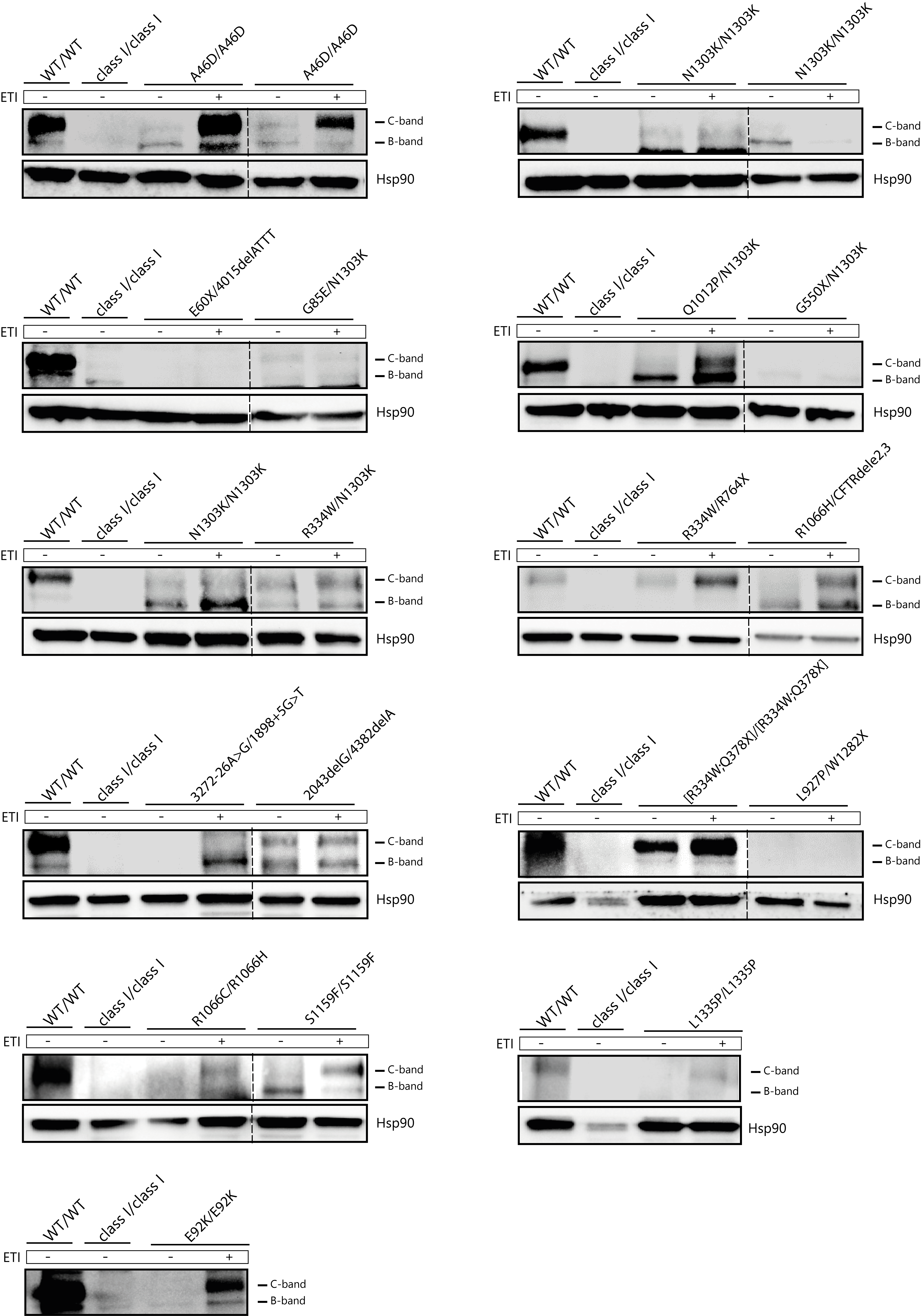

Supplement: Supplementary file 1 [file ijms-24-14539-s001.zip › SupFig1.png]

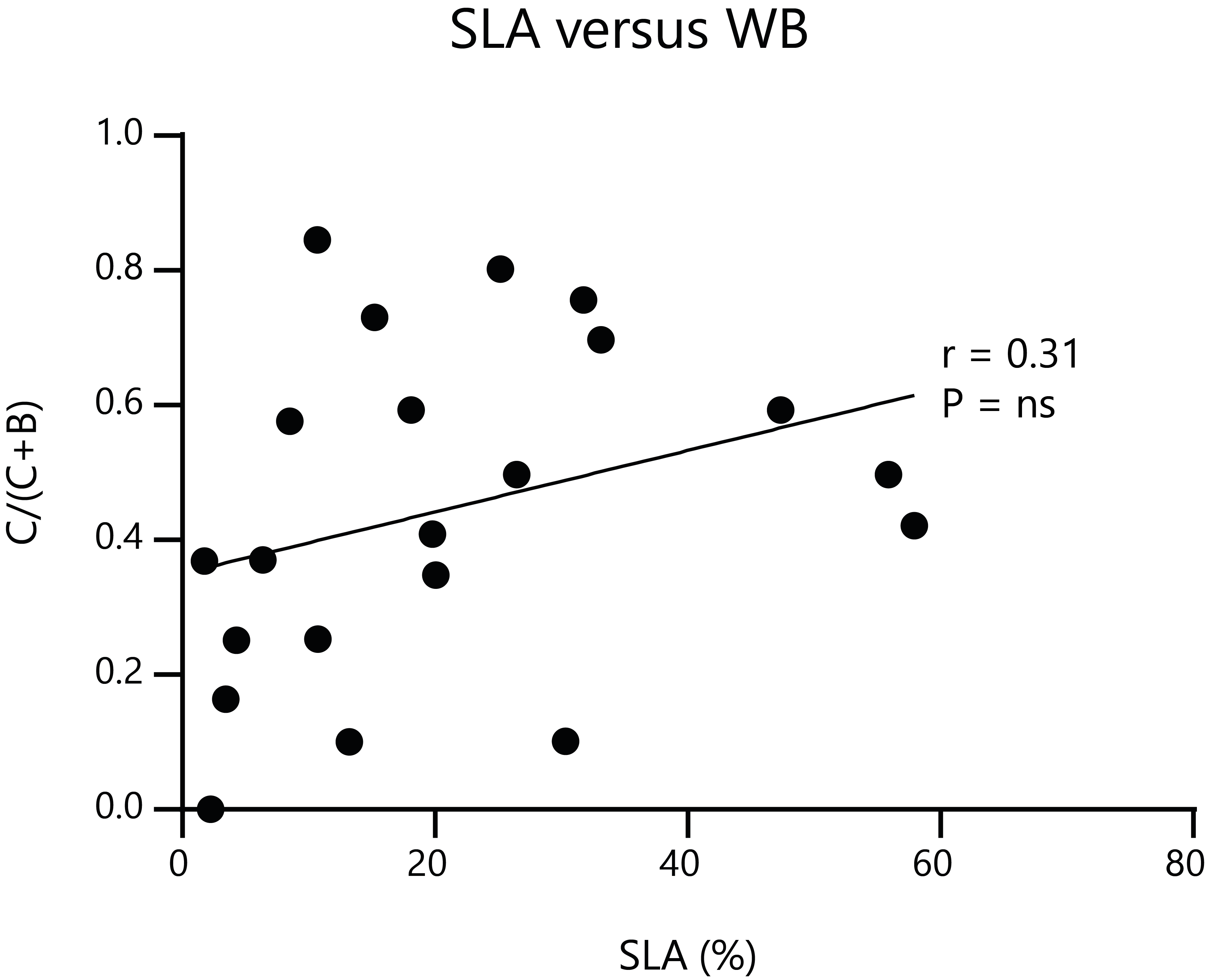

Supplement: Supplementary file 1 [file ijms-24-14539-s001.zip › SupFig2.png]

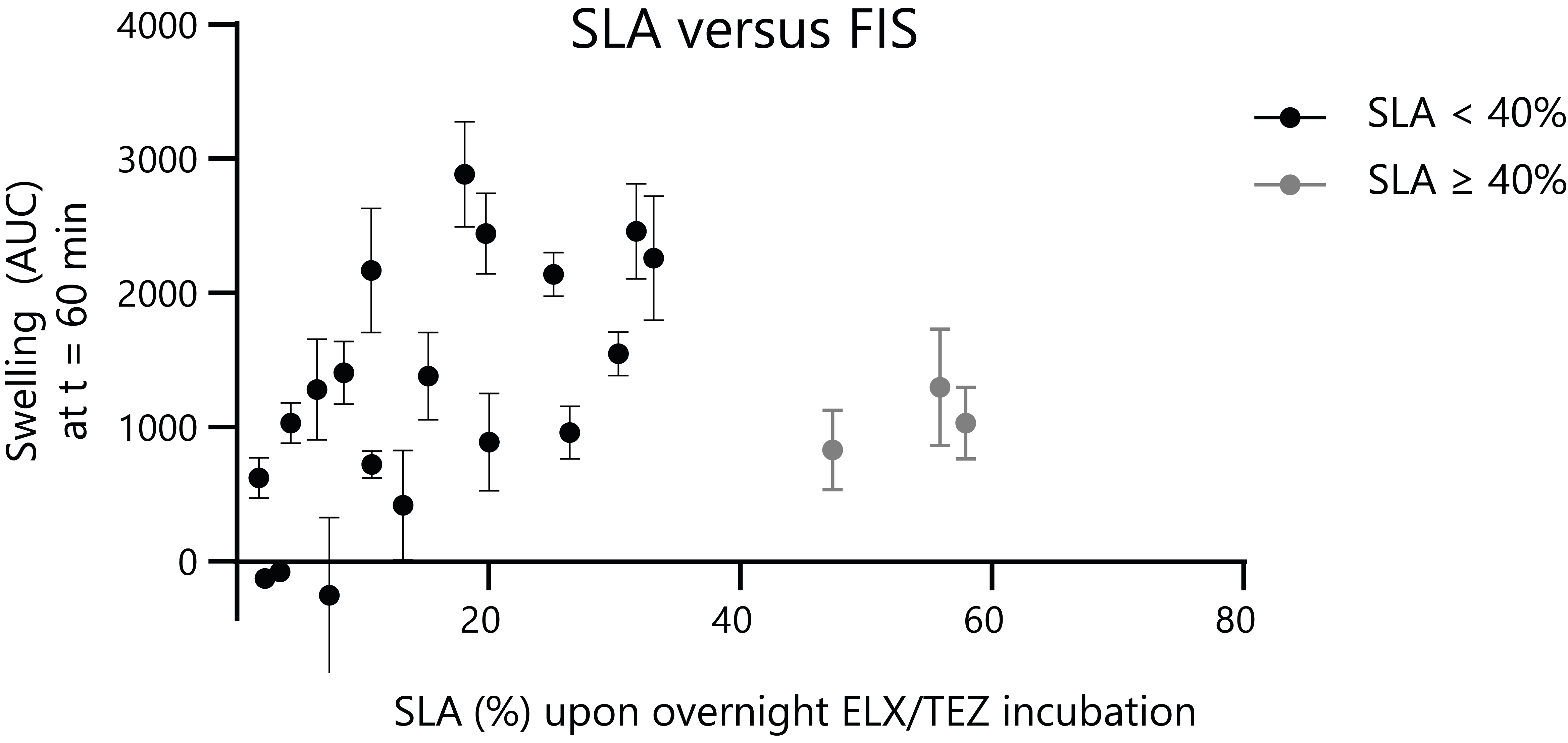

Supplement: Supplementary file 1 [file ijms-24-14539-s001.zip › SupFig4.png]
